# Management of Multi Organ Dysfunction in Neonatal Encephalopathy

**DOI:** 10.3389/fped.2020.00239

**Published:** 2020-05-15

**Authors:** Mary O'Dea, Deirdre Sweetman, Sonia Lomeli Bonifacio, Mohamed El-Dib, Topun Austin, Eleanor J. Molloy

**Affiliations:** ^1^Discipline of Paediatrics, Trinity College, The University of Dublin, Dublin, Ireland; ^2^Paediatric Research Laboratory, Trinity Translational Institute, St. James' Hospital, Dublin, Ireland; ^3^Neonatology, Coombe Women and Infant's University Hospital, Dublin, Ireland; ^4^National Children's Research Centre, Dublin, Ireland; ^5^Paediatrics, National Maternity Hospital, Dublin, Ireland; ^6^Department of Pediatrics, Stanford University School of Medicine, Stanford, CA, United States; ^7^Brigham and Women's Hospital, Harvard Medical School, Boston, MA, United States; ^8^Neonatal Intensive Care Unit, Cambridge University Hospitals NHS Foundation Trust, Cambridge, United Kingdom; ^9^Neonatology, Children's Hospital Ireland (CHI) at Crumlin, Dublin, Ireland; ^10^Paediatrics, CHI at Tallaght, Tallaght University Hospital, Dublin, Ireland

**Keywords:** Neonatal Encephalopathy, Therapeutic hypothermia, brain injury, multi-organ dysfunction, neurodevelopmental outcome

## Abstract

Neonatal Encephalopathy (NE) describes neonates with disturbed neurological function in the first post-natal days of life. NE is an overall term that does not specify the etiology of the encephalopathy although it often involves hypoxia-ischaemia. In NE, although neurological dysfunction is part of the injury and is most predictive of long-term outcome, these infants may also have multiorgan injury and compromise, which further contribute to neurological impairment and long-term morbidities. Therapeutic hypothermia (TH) is the standard of care for moderate to severe NE. Infants with NE may have co-existing immune, respiratory, endocrine, renal, hepatic, and cardiac dysfunction that require individualized management and can be impacted by TH. Non-neurological organ dysfunction not only has a negative effect on long term outcome but may also influence the efficacy of treatments in the acute phase. Post resuscitative care involves stabilization and decisions regarding TH and management of multi-organ dysfunction. This management includes detailed neurological assessment, cardio-respiratory stabilization, glycaemic and fluid control, sepsis evaluation and antibiotics, seizure identification, and monitoring and responding to biochemical and coagulation derangements. The emergence of new biomarkers of specific organ injury may have predictive value and improve the definition of organ injury and prognosis. Further evidence-based research is needed to optimize management of NE, prevent further organ dysfunction and reduce neurodevelopmental impairment.

## Introduction

Neonatal encephalopathy (NE), is a clinically defined syndrome of disturbed neurologic function in the earliest post-natal days of life in an infant born at or beyond 35 weeks of gestation, manifested by a subnormal level of consciousness or seizures, and often accompanied by difficulty with initiating and maintaining respiration and depression of tone and reflexes (1). In up to 50% of cases of NE the exact underlying cause is unknown and is commonly a combination of factors (2). The terms NE and hypoxic-ischaemic encephalopathy (HIE) are used interchangeably in the literature but as NE is all-encompassing and does not specify etiology, it is the term used in this review (3–5).

The neonatal period is the highest risk for brain injury during the lifespan. In 2010, the estimated global burden of NE was 1.15 million, with 96% of these infants being born in low and middle income countries (6). The global estimated incidence from a systematic review in 2010 is 8.5 per 1,000 live births, with an estimated incidence of 1–3 per 1,000 births in high income countries.

Therapeutic hypothermia (TH) is known to be neuroprotective by addressing the cascade of injurious events that follow a hypoxic-ischemic insult in NE. Randomized controlled trials demonstrated the safety and the efficacy of TH by demonstrating a reduction in death and major neuro-disability for infants with moderate to severe NE when their clinical history, laboratory criteria, and neurological exam meets agreed standardized criteria. Despite TH, the incidence of death or moderate/severe disability remains high at 48% (7). A systematic review including seven randomized controlled trials (RCT) with 1,214 neonates with NE undergoing TH concluded an overall mortality of 28%, with a range of 24–38% (7), with the following incidences of neurological impairment; cognitive impairment 24%, cerebral palsy 22%, epilepsy 19%, and cortical visual impairment 6%.

These high morbidity and mortality rates suggest that there is a need to improve outcomes by further optimizing TH candidate selection, improving timeliness of treatment initiation, increasing the use of brain monitoring for the identification and treatment of seizures, improving multi-organ management during TH, and identifying biomarkers to offer individualized adjunctive therapies during TH. There is no single gold standard diagnostic test to determine etiology, severity, or prognosis at present (8, 9).

Neurological dysfunction is only part of the spectrum of injury in NE following hypoxic ischemic insult, infants can have co-existing multi-organ dysfunction which contributes to subsequent morbidities and mortality. The pathophysiology underlying the brain injury in NE affects the immune, respiratory, endocrine, renal, hepatic, and cardiac functioning (10, 11). In addition, exposing these infants to TH has its own impact on multi-organ function. Optimisation of multi-organ monitoring and support during TH has the potential to prevent injury progression and enhance the neuroprotective effects of TH.

## Therapeutic Hypothermia

TH is the standard of care for moderate and severe NE. Major randomized clinical trials (RCT) have demonstrated a reduction in death and disability with TH (12). A Cochrane meta-analysis of these trials concluded that in infants over 35 weeks and <6 h of age with moderate or severe NE (*n* = 638), TH to 33.5–34.5°C for 72 h, reduced the mortality and disability at 18 months of age with a typical risk ratio (RR) of 0.75 with a number needed to treat (NNT) of 7 (12). The National Institute of Child Health and Human Development (NICHD) and Committee of the Fetus and Newborn of the American Academy of Pediatrics (AAP) subsequently published a framework to ensure the appropriate use of TH (13), and recommended that infants should meet inclusion criteria outlined in clinical trials as follows: gestational age >36 weeks; age <6 h; pH of ≤ 7.0 or a base deficit of ≥16 mmol/L in of umbilical cord blood or blood obtained during the first hour after birth; history of an acute perinatal event; 10 min Apgar score of <5 or assisted ventilation at birth and for 10+ min; neurologic examination demonstrating moderate to severe encephalopathy.

Evidence from animal studies suggest that earlier TH initiation increases neuroprotection (14). The Neonatal Resuscitation Programme (NRP) notes however that there is a paucity of evidence regarding commencement of hypothermia during resuscitation, as passive hypothermia without core temperature monitoring during resuscitation may result in overcooling with serious adverse effects (15). Thoresen et al., showed significantly improved motor development scores at 18 months when TH is initiated <3 h (*n* = 35) compared to >3 h of age (*n* = 30) (16). This suggests that once indicated, TH should be initiated immediately after resuscitation.

Two further RCT examined modifications from the original TH protocols. Laptook et al. (17) examined late initiation of TH in NE at between 6 and 24 h of age. The trial was a multicentre RCT (*n* = 164) comparing late initiation of TH with normothermia. Although there was no statistically significant difference in death and disability between groups, the authors used a pre-specified Bayesian analysis to demonstrate that there was an increased probability of reduction in death or disability with minimal adverse events in the TH group. Shankaran et al. (18) examined TH to a lower temperature of 32°C and/or for a longer duration of 120 h in a multi-center RCT. The trial was stopped at 50% (*n* = 364) of the planned recruitment due to a number of adverse events including anuria, arrhythmia, increased inhaled nitric oxide requirement and extra corporeal membrane oxygenation (ECMO) use, more days of oxygen and a higher incidence of bradycardia as well as trend toward increased mortality. There was no difference between treatment groups in the primary outcome of death or neuro-disability at 18–22 months of age.

The initial TH protocols included infants with moderate to severe NE. Despite recommendations from organizations like the AAP, there is evidence of therapeutic drift with TH being provided to neonates with mild NE. A national survey in the UK reported that 75% of centers offered TH to infants with mild NE (19). The Prospective Research on Infants with Mild Encephalopathy (PRIME) Study found that 52% of infants with mild NE had an abnormal early aEEG or seizures, abnormal brain MRI, or neurological exam at discharge (20). At follow up at a mean of 19 months, 16% of infants had a disability and 40% had Bayley III scores <85 (21). Murray et al. noted that children with mild NE, not treated with TH, had cognitive outcomes similar to that of children with moderate NE, who were treated with TH (22). A systematic review examining outcome of infants with mild NE found that 25% (*n* = 341) had abnormal neurodevelopment outcome (23). There is an ongoing phase two RCT by Thayyil et al., Optimizing the duration of Cooling in Mild NE (COMET Study; NCT03409770), examining the feasibility and duration of TH in mild NE in the UK. Interpretation of research and smaller cohort studies of neonates with mild NE is limited by a lack of consistent definition of mild NE across study groups. Although a beneficial effect of TH in this population is plausible, to date this benefit has not been demonstrated in an RCT (24).

## Neurological Assessment and Monitoring

### Neurological Assessment

A detailed neurological assessment is required to diagnose NE and ideally before the administration of sedating medications that may alter the neurological examination. The Modified Sarnat Examination (25) is used in the evaluation of neurological status for initiation of TH. This was used as entry criteria in the main TH RCTs (26) apart from the Total Body Hypothermia (TOBY) trial and the Cool Cap Study, both of which included amplitude integrated electroencephalogram (aEEG) as an entry criterion (27). The Sarnat Score assesses three stages of NE: mild (NE I), moderate (NE II), and severe (NE III) which were correlated with clinical outcome. The score is derived from a study of 21 infants with NE graded according to their level of encephalopathy and EEG findings over the first post-natal week of life. Sarnat et al. concluded that persistence of moderate encephalopathy for more than 7 post-natal days was associated with poor neurologic outcome or death (25). A systematic review of newborn assessment to predict neurological outcome including 12 studies concluded that the risk of neonatal death was 24-fold higher in Sarnat III than Sarnat II, and 171 times higher in Sarnat III than Sarnat I (28).

The Amiel-Tison Neurologic Assessment at Term (ATNAT) (29) was developed to provide a framework for observing the development of cortical control in infants at term and has been shown to predict the occurrence of cerebral palsy after birth asphyxia. Amess et al. examined term infants postnatally (*n* = 28) with NE with the ATNAT at 8 h and at 7 days. Both early and late neurological examinations were reliable indicators of a favorable outcome at 1 year, having negative predictive values of 100 and 91%, respectively (30). Murray et al., used the ATNAT serially over the first 3 days of post-natal life in 57 infants with NE, and found a significant correlation between ATNAT and neurological outcome at 2 years (31). A normal early assessment predicted a normal outcome, and a persistent neurological abnormality on day of life 3 was associated with neuro-disability. The risk was 100% in those with a severely abnormal ATNAT, and 41% in those with a moderately abnormal assessment.

The Thompson score is a clinical tool assessing central nervous system dysfunction in NE, based on the longitudinal clinical assessment of 9 signs, including tone, level of consciousness, seizures, posture, Moro, grasp, suck, respiratory function, and anterior fontanelle tension (32). Kothapali et al. showed that the Thompson score has a good short-term predictive capacity of morbidity and mortality (*n* = 145) (33). Mendler et al. showed a strong association between Thompson score and long-term, at a mean age of 53 months in infants with NE (*n* = 36) (34). Almost all surviving infants with a maximal Thompson score ≤ 10 had a normal IQ and almost all infants with an impaired IQ (<85) had a Thompson Score ≥11.

Prechtl's assessment of general movements assesses spontaneous motor activity and has proven sensitive in the prediction of cerebral palsy (35). General movements assesses for two distinct patterns of normal movements, writhing movements at 6–9 weeks post term, and fidgety movements at 6–20 weeks. Abnormal movements include poor repertoire, cramped-synchronized, and absent fidgety general movements. General movements were highly correlated to gray matter injury in infants with NE (36). All infants with severely abnormal general movements had gray matter injury and poor motor outcome. Infants with predominantly white matter, cortical lesions or mild basal ganglia or thalamic injury had normal or transiently abnormal general movements and normal or mild motor impairment. The ATNAT was compared to Prechtl's qualitative assessment of general movement in a group of 45 preterm infants with risk factors for brain injury and correlated better with neurodevelopmental outcome (37).

## Amplitude Integrated EEG (AEEG) and Identification and Management of Neonatal Seizures

The incidence of seizures in NE is ~50% (38). The presence of seizures increases the incidence of neurodevelopmental impairment. A cumulative seizure burden of 40 min increases neurodevelopmental impairment by nine-fold, independent of grade of encephalopathy and TH (39). TH reduces the seizure burden, especially in moderate NE (40). Seizures can be difficult to diagnose in neonates as ~50% do not have obvious clinical signs (41, 42).

## Amplitude Integrated EEG (AEEG)

Availability of continuous Electroencephalography (EEG) with real-time interpretation by trained staff is ideal but most NICUs use the modified form of amplitude integrated EEG (aEEG), a single or double lead EEG recording from two parietal electrodes. aEEG is useful to monitor baseline brain activity and to detect seizures (43). The combination of early neurological examination and aEEG, in comparison to each individually enhances the ability to identify infants with NE (44). Svenningsen et al. demonstrated that aEEG background activity over the first 6 h of post-natal life accurately predicted neurological outcome (45). The limitations of aEEG include limited short low voltage seizure detection (46) and inaccurate readings due to artifact compared to EEG. aEEG has been demonstrated to be less predictive of outcome at early time points in infants treated with TH compared to normothermia; infants with good outcome had normalized background pattern by 24 h when treated with normothermia and by 48 h when treated with hypothermia (47). Various aEEG training modalities are available such as from the Total Body Hypothermia Register website (https://www.npeu.ox.ac.uk/downloads/files/toby/TOBY-CFM-Manual.pdf).

## Treatment of Neonatal Seizures

The treatment of seizures in NE remains a therapeutic challenge (48). Phenobarbitone is used as first line agent (49), however its efficacy is limited. Phenobarbitone has been demonstrated to reduce both the amplitude and propagation of seizures (50) which may result in electroclinical uncoupling of seizures, which refers to electrographic seizure activity that is not clinically manifested and may make seizure detection more difficult on aEEG monitoring. There are concerns about adverse cognitive effects of phenobarbitone on the neonatal developing brain (51). Animal models have demonstrated that early phenobarbitone exposure causes adverse neurological outcomes later in life (52) and most of the pharmacological data on phenobarbitone is extrapolated from adult data (53). The “efficacy of intravenous levetiracetam in neonatal seizures” trial (NEOLEV2; NCT01720667) examined the efficacy of using levetiracetam first line in comparison to phenobarbitone for neonatal seizures from all causes. The primary outcome was to determine the efficacy of intravenous levetiracetam in terminating neonatal seizures (from all causes) when given as first line therapy compared to phenobarbitone. The provisional results demonstrated that phenobarbitone was more effective then levetiracetam with 80% remaining seizure free for 24 h compared to 28% (54). Sharpe et al. provided continuous monitoring for patients in the NEOLEV2 trial with real-time response to seizure detection. They reported that automatic seizure detection algorithm was useful but not accurate enough to replace human review and that placement of EEG monitors after hours was problematic (55). Levetiracetam pharmacokinetic studies in term neonates found a higher than expected renal clearance, that increased significantly over the first week of post-natal life requiring increased interval dosing (56).

Second line agents to treat neonatal seizures include phenytoin, levetiracetam, topiramate, lidocaine, and midazolam (57). Phenytoin was found to be 57% effective as a combination therapy with phenobarbitone to achieve seizure control in an RCT of neonates with seizures from all causes (*n* = 59) (53). Bumetanide was evaluated as a second line therapy for neonatal seizures via the NEMO (Treatment of Neonatal seizures with Medication Off-patent: evaluation of efficacy and safety of bumetanide) open label feasibility trial. The trial had safety concerns regarding ototoxicity and was stopped prematurely (*n* = 14) (58). Bumetanide did not show signs of clinical efficacy for the treatment of neonatal seizures in the 14 infants that were studied prior to the premature cessation.

A survey of 55 pediatric neurologists predominantly based in USA showed a high off label use of levetiracetam and topiramate as second line for neonatal seizures (3). No side effects in the levetiracetam group were reported in the survey and the treatment was reported as beneficial in over half of cases. There was no consensus on the dosage to use and a wide range of doses were reported (10–30 mg/kg). Midazolam has been shown to have a good response rate in seizure termination in a group of neonates with seizures refractory to phenobarbitone and phenytoin (59). A retrospective review of neonatal seizure management reported that lidocaine demonstrated effectiveness in 50% of neonatal seizures as second line therapy but caution is warranted in view of cardiac toxicity, with bradycardia reported in 3% of patients (60). These studies demonstrate that there is an urgent need for clinical trials to determine safe and effective treatment for neonatal seizures (61).

In summary, 50% of infants with NE have seizures, with phenobarbitone remaining the first line agent with demonstrated clinical efficacy but some overall safety concerns. A global working group of experts established a consensus for protocols for new RCTs in neonatal seizures using evidence based neonatal seizure treatment (62).

## Neuro Imaging

MRI is the gold standard technique to detect patterns of cerebral damage in NE and provides a reliable guide to prognosis (63). Cranial ultrasound (US) and Doppler sonography have useful adjuncts in early diagnostic imaging (64–66). Conventional MRI, with T1 and T2 weighted images, has been demonstrated to have good diagnostic ability to detect brain injury at the end of the first post-natal week of life. There are limitations to the predictive value of MRIs, a normal MRI does not guarantee a normal neurodevelopmental outcome. In one study 32% of infants who had a normal MRI brain in the neonatal period post TH had abnormal development at follow up (67).

The advancement of MR diffusion weighted imaging (DWI) (68) and magnetic resonance spectroscopy (MRS) means lesions may be visualized within the first few post-natal days.

The optimal timing of the MRI brain is important for clinical prognostication and potential redirection of care in the case of end of life decisions, with most cases of withdrawal of care occurring in the first three post-natal days of life (69).

High correlation of sequential conventional MRI and DWI on post-natal day 4 and during the second week was demonstrated in 15 patients with NE (70). 3 Tesla (T) MRI brain was performed in 12 infants with NE at four time points, on post-natal day 1, days 2–3, days 8–13, and at 1 month of age (71). All injuries were already visible on early MRI scans, the later MRIs did not show any new lesions, and in severe NE, DWI changes were subtle on post-natal day 1 and became more apparent on days 2–3. The image quality and diagnostic accuracy in comparison is better with 3T in comparison to a conventional 1.5 T MRI (72). 3T MRI has a good safety profile and is safe for the developing brain but is not universally available (73).

MRI changes in NE may commonly involve parasagittal watershed infarcts between anterior/middle cerebral artery and middle/posterior cerebral artery, with both cortical and subcortical involvement, and injury to metabolically active tissues such as the basal ganglia (BG), thalami, putamen, hippocampi, brainstem, and corticospinal tracts (74). Abnormalities in the signal of the posterior limb of the internal capsule (PLIC), BG and thalami have been demonstrated to have the greatest predictive value of poor neurodevelopmental outcome, in particular for motor outcome (75, 76). Severe BG and thalami lesions predictive accuracy for severe motor impairment was 0.89 in one study, with abnormal PLIC signal intensity predicting the inability to walk independently by 2 years with a positive predictive value of 0.88 (77). In contrast, infants with white matter damage and no BG or thalamic injury had a good prognosis for independent walking by 2 years of age in infants with NE (*n* = 270) (78).

TH has demonstrated a reduction in BG, thalamic, white matter, and PLIC signal abnormalities (79). TH may be better at reducing the severity of BG and thalamic injury as these regions are selectively vulnerable to acute hypoxia-ischemia compared to parasagittal areas which are more associated with partial prolonged injury (80). The accuracy of MRI as a biomarker to predict outcome is not altered by TH (79).

Magnetic resonance spectroscopy (MRS) provides a non-invasive examination of biochemical brain biomarkers. The Magnetic Resonance Biomarkers in Neonatal Encephalopathy (MARBLE) Study (81) found that thalamic N-acetyl aspartate (NAA) concentration had the highest sensitivity (100%) and specificity (97%) to predict neurodevelopmental outcome at 2 years (*n* = 223). A meta-analysis on MR biomarkers identified MRS deep gray matter Lactate/NAA as the most accurate biomarker to predict neonatal outcome and commented that MRS scoring systems can increase prognostic objectivity (82).

Diffusion tensor MRI is more sensitive than conventional MRI to explore brain development and white matter fibers density and maturation (83), and can display early injury prior to T1 and T2 abnormalities being apparent (84). A systematic review by Dibble et al., of white matter tracts in NE, found three areas of altered diffusion commonly seen in NE were associated with adverse outcomes, the posterior limb of internal capsule and the genu and splenium of the corpus callosum (85). Gray matter diffusion changes in the BG and thalami post NE are associated with dyskinetic cerebral palsy (86). Weeke et al. included diffusion weighted changes in the corpus callosum in a new NE MRI brain scoring system correlating with outcome at 2 years (87). The limitation however of DWI is that the changes normalize within the first week (88).

In addition to conventional MRI techniques, there are further advanced research methods where the acquired data is amenable to derive specific measures through computational analysis and exploration of their ability to predict outcome. This includes diffusion MRI tractography to visualize white matter structure (89–91), and model based measures of tissue microstructure such as Neurite Orientation Dispersion and Density Imaging (NODDI) (92, 93) and diffusion tensor imaging (DTI) based parameters like apparent diffusion coefficient (ADC), radial diffusivity (RD) and fractional anisotropy (FA) (94). Other physiological parameters such as regional cerebral blood flow can be measured with Arterial Spin Labeling (ASL) (95).

Near-infrared spectroscopy (NIRS) is a tool to monitor regional cerebral oxygen saturation, via a calculation based on the absorption spectra of oxygenated and deoxygenated hemoglobin. The measurement of regional cerebral oxygen saturation represented mixed oxygenation of both arterial, venous and capillary readings (96). A systematic review of the use of NIRS in NE showed an association between impaired cerebral autoregulation and cardio-respiratory injury, abnormal MRI and long-term outcome (97).

## Neurocritical Care

Neurocritical care is an evolving field of tertiary intensive care units through collaboration of neonatologists, neurologists, nurse specialists, and allied health professionals who coordinate care for neurologically ill neonates and has improved outcome of babies with NE (98).

Neurocritical care provides consistency in diagnostic and management strategies focused on improved neurological outcomes. A retrospective review compared the MRI brains' of infants cared for in their normal NICU (*n* = 109) to those of infants cared for after their introduction of a neurocritical ICU (*n* = 107) (99) and demonstrated a reduction in abnormalities on MRI brain of infants cared for in the neuro NICU after adjusting for confounding factors (odds ratio 0.3, CI 0.15–0.57, *p* < 0.001). The changes implemented in that neuro NICU included the introduction of a new multi-disciplinary team, full EEG monitoring done for duration of hypothermia and rewarming, neuroprotection protocols, quality improvement practices, and implementation of a long-term follow-up program.

## Cardiovascular

The spectrum of cardiovascular (CVS) dysfunction in NE ranges in severity and may be attributed to hypoxia or be secondary to ischemia, metabolic acidosis, and multiorgan injury (100). Myocardial contractility, cardiac output and blood pressure are all negatively impacted and co-existing pulmonary hypertension is common. CVS dysfunction may be evaluated from a number of modalities including vital signs, biochemical parameters, echocardiography, and other haemodynamic assessments.

TH affects haemodynamic functioning by causing bradycardia (101), peripheral vasoconstriction, and decreasing cardiac output (102). TH increases the QT_c_ interval (103) and increases the risk of cardiac arrhythmias (104). The initial TH RCTs were not adequately powered to examine cardiovascular benefit however creatinine-kinase muscle/brain (CK MB) and brain natriuretic peptide (BNP) decrease with TH suggesting a cardioprotective effect (105) and animal model studies have demonstrated an improvement in cardiac ischaemia (106).

Both troponin-T and troponin-I have been demonstrated to be sensitive markers of cardiac dysfunction in NE (100, 107). Gunes et al. measured serial troponin-T, creatinine Kinase (CK-MB), in 45 infants with NE (108). CK-MB levels were significantly higher in moderate and severe NE then in mild NE. Boo et al. found the sensitivity of serum troponin-T in detecting myocardial injury in NE presenting with heart failure was 72.7% and the specificity was 35.9% (109). Serial serum troponin levels during the first 48 h of post-natal life have been found to be significantly higher in infants with NE who died.

Hypotension is observed in up to 62% of patients and may cause secondary multiorgan ischaemic injury. There is no consensus on the ideal target mean systolic, diastolic nor pulse pressure during TH nor on the best pharmacologic agents to maintain it. Long term clinical outcomes of inotropic and chronotropic support lack evidence, with dopamine, dobutamine, and adrenaline commonly chosen demonstrating short term haemodynamic improvements in BP (110). Vasopressor use warrants caution due to potential pulmonary and systemic vasoconstriction (110). McNamara et al. recommended that inotropes administration be done on an individual patient basis depending on echocardiography (ECHO) and clinical status (111). Echo is the best diagnostic tool available to assess cardiovascular function and guide inotropic, chronotropic, and fluid management.

Medications used to treat systemic hypotension include dopamine, dobutamine, adrenaline and noradrenaline, depending on coexisting myocardial dysfunction. Adrenaline may be the most appropriate inotrope due to its' action on α_1_, α_2_, β_1_, and β_2_ receptors and its' favorable impact on pulmonary vascular resistance (PVR)/systemic vascular resistance (SVR) ratio (112). The action of dobutamine via α and β receptors decreasing SVR may have advantages as an inotrope in the context of persistent pulmonary hypertension of the newborn (PPHN) and myocardial dysfunction but has not been subject to controlled trials (113). Dopamine is predominantly a vasopressor and in neonatal animal studies has been shown to increase PVR and SVR (112), which has the potential to increase afterload, decrease left-to-right shunting, and compromise systemic oxygen delivery (113). Dopamine is the most widely studied and the most commonly prescribed inotrope in neonatology (114). Studies of developmental outcome favor dobutamine use over dopamine in the preterm population but there are no comparative RCTs in NE (115). McNamara et al. recommended dobutamine use in NE to improve cardiac contractility and heart rate. Milrinone has altered pharmacokinetics during TH affecting its clearance and caution needs to be used with noradrenaline administration as there is little evidence of benefit and it has not been subject to RCT for follow up data (111).

A sustained difference of >5–10% between continuous pre- and post- ductal saturation monitoring may indicate PPHN, which can be confirmed on echocardiography (116). Echocardiography helps to quantify the degree of PPHN and guides treatment including choice of inotropic support. The development of serial functional echocardiography in the NICU allows tracking of the dynamic changes occurring over the course of PPHN (113, 117).

The management of PPHN involves reducing the cardiac afterload and maintaining high preductal mean blood pressures (113, 118). Milrinone has been shown to improve oxygenation index in term neonates with severe PPHN without compromising systemic blood pressure (119). A Cochrane review (120) concluded that the efficacy and safety of milrinone in the treatment of PPHN are not known and recommended that use is restricted to RCTs. Milrinone metabolism is known to be decreased by TH and in an animal model study the inotropic effect was abolished at temperatures of between 31 and 34°C (121). Sildenafil is increasingly used in PPHN. A Cochrane review of sildenafil for PPHN found a significant reduction in mortality in the sildenafil group vs. the control group with a number needed to treat of three (122). The review concluded that sildenafil has significant benefits especially in resource-limited settings and recommended a large-scale randomized control trial comparing sildenafil to the currently used vasodilator inhaled Nitric Oxide (iNO). A Cochrane Review (123) of iNO for respiratory failure in near term or term infants found that iNO improved the outcome in hypoxaemic term infants by reducing the incidence of the combined endpoint of death or need for extra-corporeal membrane oxygenation (ECMO). Oxygenation improved in ~50% of infants receiving iNO. Long-term follow up studies have found no increase in neurodevelopmental impairment with its use (124, 125).

In summary, cardiovascular dysfunction in NE can be negatively impacted by TH. Echo is the best tool to guide management and other physiological parameter thresholds have not been well-defined. Inotropic and chronotropic medications have altered pharmacokinetics during TH and the choice of agent is best guided by individual hemodynamics.

## Respiratory Management in NE

The incidence of respiratory dysfunction in babies with NE varies from 23 to 86% (10, 11, 126, 127). The spectrum of injury ranges from transient oxygen requirement to severe persistent pulmonary hypertension (PPHN). The pathogenesis of pulmonary dysfunction is complex, although hypoxia is a major component via disruption of the normal physiological fall in pulmonary vascular resistance (113, 118, 128, 129).

The Neonatal Resuscitation Program^®^ (NRP), 7th edition (130) and European Resuscitation Council Guidelines (131) advise on initial management and resuscitation for a non-vigorous term infant. The NRP recommends resuscitation using 21% fraction of inspired oxygen concentration and titrating the oxygen to maintain oxygen saturations within a standardized oxygen centile range depending on minutes of life. The exception to this is if a neonate requires cardiopulmonary resuscitation to titrate the fraction of oxygen to 100%. Neonates are at risk of hyperoxia when exposed to high oxygen concentration, after coming from a relatively hypoxic environment *in utero* and their free radical scavenger systems are underdeveloped (132). Infants with perinatal stress (*n* = 609) were enrolled in a multi-center RCT comparing resuscitation at 100% fraction of inspired oxygen to 21% (133). The infants with higher oxygen exposure had more oxidative stress but no differences were found in mortality or short-term morbidity. This study was limited by the fact that the infants who were resuscitated were relatively well, as all had oxygen saturations of over 90% at 2 min and <2% of them required supplementary oxygen after resuscitation. Expert opinion from this study recommended restoring normoxia as quickly as possible during resuscitation and that a properly powered RCT to establish correct fraction of inspired oxygen would need to recruit 7,000 neonates (134).

Respiratory support in NE aims to maintain a pH over 7.25 and a normal to high partial pressure of arterial carbon dioxide (PaCO_2_ 5–7 kPa, 37.5–52.5 mmHg) (113, 135–137). Hypocarbia has detrimental effects on cerebral perfusion in an already compromised infant (138–143) and is associated with neurosensory hearing impairment and abnormal neurodevelopment (143, 144). Both isolated low PaCO_2_ levels and cumulative PaCO_2_ <4.6 kPa (35 mmHg) were associated with death and disability.

Hypothermia is known to decrease the partial pressure of oxygen and carbon dioxide whilst increasing the pH (142, 145–147). The temperature corrected blood gas values can be obtained by inputting the temperature to the blood gas analyser. Fraction of inspired oxygen, mean airway pressure, oxygenation index, and alveolar-arterial gradient decrease during induction of TH increase during rewarming. Minute ventilation increases with TH and decreases upon rewarming. The inspiratory time, respiratory rate, and positive end expiratory pressure are unaffected. Eicher reported a higher iNO requirement with TH, with 5/35 neonates requiring iNO compared to 1/30 managed at normothermia (*p* < 0.01) (148), however a Cochrane meta-analysis of four TH trials showed no significant effect of hypothermia on PPHN (26).

In summary TH impacts respiratory function in neonates and requires altered blood gas interpretation. Tight control of carbon dioxide and avoidance of hypoxia is essential. There is expert evidence on the use of iNO in PPHN however trials to evaluate the evidence of milrinone and sildenafil use are required.

## Renal Management in NE

Renal dysfunction resulting in acute kidney injury (AKI) varies from 22 to 70% in NE (149). Selewski (150) reported that infants with NE and co-existing AKI had both a longer length of stay even after controlling for other confounders and an increased incidence of abnormal MRI brain (151). TH has not been associated with a reduction in AKI in NE (150).

The current Kidney Disease Improving Global Outcomes (152) (KDIGO) guidelines for AKI use a rise in creatinine as part of its definition. Creatinine is not an ideal biomarker of neonatal AKI as it peaks late, only rises when 50% of renal function is impaired, may reflect maternal creatinine level, and reflects kidney function rather than injury.

The optimal biomarker would diagnose AKI earlier so active management can be initiated. Cystatin C is a better indicator of glomerular filtration rate than creatinine and correlates with NE severity (153, 154). Neutrophil gelatinase- associated lipocalin (NGAL) correlates to severity of NE and can predict a later creatinine rise (155). Neonates with moderate to severe NE had significantly elevated urinary levels of cystatin-C, NGAL and lower epidermal growth factor in comparison to mildly affected infants (156).

Electrolyte abnormalities were seen in 50% of infants, with hyponatraemia, hypokalaemia, and hypocalcaemia being the most common (157). Renal profile, fluid balance, urine electrolytes, and acid-base balance need regularly monitoring. Urinary catheterisation may be necessary as morphine can cause urinary retention via anti-cholinergic effects.

Oliguria is common in NE. There is a significant risk of fluid retention and hyponatremia due to a poor capacity to produce urine. Fluid intake is frequently restricted in NE during TH due to concerns regarding cerebral oedema (158). A Cochrane review however found no RCT evidence to support this practice and recommended further studies (158). A subsequent RCT of infants with NE undergoing TH randomized infants to a restricted fluid intake of two thirds of normal (*n* = 40); at 40, 55, 65, and 80 mls per kilogram per day on post-natal days one to four of life, respectively, vs. normal fluid intake (*n* = 40); of 60, 80, 100, and 120 mls per kilogram per day, respectively, for the first four post-natal days of life. The fluid composition was 10% dextrose in the first 48 h of post-natal life with sodium and postassium added to the dextrose over the next 48 h. Restricted fluid did not reduce death or major neuro-disability at 6 months of age and was associated with a trend toward more hypoglycaemia (159). Hyponatraemia can result from kidney injury causing fluid retention, the syndrome of inappropriate anti diuretic hormone (SIADH), and tubular dysfunction. The Bartter and Schwartz (160) criteria define SIADH as hyponatremia (serum Na^+^ <135 mmol/L) with a corresponding serum hypoosmolality (<280 mOsm/kg), and continued renal excretion of Na^+^ (>40 mEq/L), in the absence of clinical evidence of volume and of other causes of hyponatremia. Water restriction is necessary to manage the SIADH safely (161).

During TH renal perfusion is reduced, pharmacokinetic parameters change, and therefore there is a reduction in renally excreted drugs. Nephrotoxic medications, such as acyclovir, aminoglycosides, non-steroidal anti-inflammatory drugs, and vancomycin administration are recommended at renal doses and require therapeutic monitoring. Cefotaxime can be substituted for gentamicin as it has similar coverage without nephrotoxicity (162). The PharmaCool study group demonstrated that morphine-6-glucuronide, the active metabolite of morphine, excretion was decreased during TH. They recommend a loading dose of 50 mg/kg of morphine followed by 5 mcg/kg/hour during TH but acknowledged that there is a large variability of plasma concentrations between patients so dosing may require alterations on an individual patient basis (163).

Renal replacement therapy for severe AKI refractory to medical therapy is not a frequently used therapy, but when indicated peritoneal dialysis is preferred over continuous renal replacement therapy (164). There is a lack of data with long term renal follow up of neonates from a renal perspective post AKI, despite the knowledge that AKI from all causes carries the risk of chronic kidney disease (CKD) with Mammen (165) et al. finding that 10.3% of children had CKD in the 1–3 years post AKI. Askenazi et al. (149) recommend the need for post AKI long term follow up on a three monthly basis with urinalysis and blood pressure measurement identify those children who will go on to develop chronic kidney disease.

To summarize, the definition of AKI is less suitable in neonatal AKI. Electrolyte disturbance and SIADH are common in NE and require monitoring. Morphine elimination is decreased by TH. There is no consensus on AKI management in NE nor recommendation for long term follow up which is essential post AKI. The optimal fluid management was subject to a small RCT on whether to restrict fluids with no difference in outcome.

## Gastrointestinal, Liver, and Nutrition

Enteral feeds were held in the initial TH RCTs, although the risk of necrotizing enterocolitis was similar in TH and neonates managed at normothermia. Some TH centers are now implementing trophic enteral feeds of expressed breast milk (166, 167). A pilot retrospective review of 17 neonates who received minimal enteral nutrition compared to no enteral feed during TH (*n* = 17) found the enteral feeding group was associated with a reduced length of stay and time to full feeds, and did not increase feeding complications nor systemic inflammation (168).

An important finding on the review of available evidence and literature is that no trials have examined the optimal type of fluids to be used. There are no recommendations on whether TPN or dextrose plus electrolytes is the optimal fluids. In anuric renal failure, losses (30 ml/kg/day) plus urine output replacement are recommended for infusion volume (169). There is no evidence to support the use of frusemide in fluid overload in NE.

Glycaemic control is critical in NE as glycogen stores are metabolized via anaerobic metabolism commonly resulting in hypoglycaemia. Initial hypoglycaemia and subsequent hyperglycaemia are associated with poor neurological outcome (170–175). Optimal timing and intervals of glucose monitoring is unknown, however, one evidenced based recommendation is to initiate glucose infusion rate (GIR) of 6–8 mg/kg/min, with 2 mg/kg/min increases in GIR if hypoglycaemia occurs (176). Glucose monitoring recommendation is every 30–60 min until the glucose is over 2.8 mmol/L (50 mg/dl) and subsequently every 4–6 h.

Shah et al. (10) defined hepatic involvement in NE as an elevated aspartate aminotransferase (AST) or alanine aminotransferase (ALT) to >100 IU/ during the first week after birth. Transaminitis, defined as 1.5 times the upper limit of normal was reported in 80% of babies with NE by Hankins et al. (126). They suggested that elevated lactate dehydrogenase, ALT and AST to 1.5 times the upper normal level indicates liver involvement in NE (177). Severity of NE is associated with higher ALT and AST (178). Abnormalities in markers of hepatic synthetic function such as albumin and prothrombin have not been shown to correlate with severity of NE (178, 179). Management of liver dysfunction in NE remains supportive in nature, with platelet, plasma, and albumin infusions as necessary and vitamin K administration. Caution is warranted with use of hepatotoxic medications (paracetamol, ampicillin, and gentamicin). Ensuring normalization of liver function testing in the neonatal periods avoids missing underlying metabolic disorders.

In summary, glycaemic control is critical and can contribute to neurological morbidities. There is a lack of evidence from RCTs regarding enteral nutrition during TH, and optimal fluid volume and type to be infused parenterally. Liver dysfunction requires monitoring alongside caution with hepatotoxic medications.

## Hematological Assessment and Monitoring

NE is associated with elevated nucleated red blood cells, thrombocytopenia, and prolonged coagulation profile. Coagulopathy is caused by blood loss, hypoxia-ischaemia, and disseminated intravascular coagulation. The incidence of coagulopathy causing major or life threatening bleeding reported in the initial TH RCTs ranged from 3 to 12% (26). Coagulopathy was reported in 18% in the NICHD study, 19% in the Cool CAP study, and 40% in the TOBY study. There is limited data regarding recommended levels to currently transfuse neonates to overcome coagulopathic or anemic states (180). One study reported that 57% of NE infants required a blood product transfusion in the first 12 h (180).

Foreman et al. established a higher incidence of clinically significant bleeding in infants with NE with platelets below 130 × 10 ^9^/L, fibrinogen under 1.5 g/L and international normalized ratio (INR) over 2 (181). Patel et al. define haemostatic dysfunction as prothrombin time (PT) ≥18 s, platelet count <100 × 10^9^/L and/or fibrinogen <150 mg/dl (180). Hankins et al. (177) defined hematological injury as the development of early thrombocytopenia (<100 × 10^9^ per liter) in the absence of other causes, or an increase in nucleated red blood cell count to ≥26 per 100 white blood cells. A number of guidelines recommend discontinuation of TH in the case of life threatening hemorrhage and ILCOR recommend platelet monitoring but do not specify intervals nor thresholds to intervene at (182).

TH is known to slow the production of enzymes involved in the coagulation cascade (183), but has not been demonstrated to cause an increase in any major hemorrhage (184). Severe hypoxia has been shown to decrease the platelet lifespan (185), whereas hyperoxia exposure has been demonstrated to worsen platelet aggregatory response (186). Protein C, protein S, and antithrombin III were increased in 100% of infants with NE demonstrating a potential to have an increase in thromboembolic events (187). Fetal thrombotic vasculopathy is a common finding on placental pathology in NE (188).

Neonatal stroke was implicated in 4.8% of cases of NE in one study (189). Neonatal stroke as the etiology of NE has been demonstrated to have a worse long term outcome (189, 190). The stroke may be venous or arterial in nature, and of hemorrhagic or ischemic origin. Arterial ischemic stroke previously is demonstrated to have a higher incidence in the literature, however it is more common preterm infants in comparison to term infants. Radiconi et al. hypothesized the incidence of cerebral sino-venous thrombosis is underrecognized in neonates undergoing TH (191). They found 27% of neonates had cerebral sinovenous thrombosis by performing MR venography post rewarming.

Antenatal fetal, maternal, and placental risk factors are all be implicated, including placental infarction, pre-eclampsia, maternal smoking, maternal chorioamnionitis, perinatal asphyxia, resuscitation, low Apgar scores, fetal thrombophilia (for venous stroke), and congenital heart disease (192). Alongside TH if indicated in the case of stroke causing NE, the management involves treating underlying condition.

Leukocytosis has been shown to correlate to abnormal neurodevelopmental outcome (193, 194). Morkos reported that elevated neutrophil best predicted adverse neurological outcome at 1 year.

In summary, coagulopathy is common in NE at up to 40% and blood product transfusion requirement is common. Stroke may be implicated in the etiology of NE. TH impacts coagulopathy and may require discontinuation in event of life threatening hemorrhage.

## Infection

Infection and inflammation are implicated in the etiology of NE (195). Maternal chorioamnionitis is a risk factor for NE, with up to one third of placentas in NE displaying histological chorioamnionitis (179). The Vermont Oxford Network reported that 24% of cases of NE have an associated antenatal inflammatory finding (2).

TH has not been associated with a higher incidence of culture positive sepsis (26). Robertson et al. postulated that the higher mortality in a pilot RCT in Uganda in infants receiving TH (33% died) compared to normothermia (7%), may have been related to a higher incidence of sepsis during TH, but the laboratory infrastructure was lacking to support this hypothesis (196). In animal model study TH appears to be protective in gram positive infection but not gram-negative sepsis (197). This has not been studied in controlled studies in human neonates.

Broad spectrum antibiotic therapy covering gram positive, negative, and anaerobes is common practice until sepsis has been excluded, with negative blood cultures and normal infection markers of white cell count and C-reactive protein (CRP). Caution with CRP interpretation is advised as the peak value is delayed by TH (198). Group B Streptococcal (GBS) sepsis was implicated in 0.58% of cases of NE from a systematic review (199). The NE mortality was higher in cases of GBS associated NE at 21% compared to NE mortality not complicated by GBS at 13.7%. The systematic review identified that infants with NE have a 10-fold higher risk of GBS in comparison to term infants without NE (199).

A small pilot study of 16 infants with NE screened extensively for neurotropic viruses, bacteria, and protozoa, by performing bacterial cultures in blood and cerebrospinal fluid (CSF) before antibiotic treatment, and viral CSF, polymerase chain reaction (PCR) for cytomegalovirus, herpes simplex 1 and 2, Epstein-Barr virus, enterovirus, and human parechovirus (200). One case of blood culture positive bacterial sepsis and four cases of clinical sepsis were diagnosed, with no PCR positive results.

Neonatal herpes simplex virus (HSV) central nervous system (CNS) infection may be associated with neonatal seizures and present as with similar signs to NE (201). Maternal primary infection of HSV during the third trimester or maternal mucocutaneous or genital lesions raises suspicion and warrants investigation and empiric treatment in the neonate. Mucocutaneous infection has an absence of clinical lesions in 20% of cases (202) and in 80% of neonatal HSV infection there are no known maternal risk factors (202). A rapid HSV Swab PCR testing and viral culture with the areas of skin swabbing to include the anus, conjunctivae, mouth, nasopharynx, and any suspected vesicles is advised with clinical suspicion (201). Renal function monitoring and adequate hydration are important in view of nephrotoxicity associated with acyclovir (203).

In summary, sepsis evaluation and broad spectrum antibiotics are routine in NE but there is no expert or evidence-based consensus on indication to sample cerebrospinal fluid (CSF) nor viral and bacterial PCR screening. The incidence of HSV and GBS is higher in NE.

## Skin

Regular full skin observation is suggested in view of the potential complications during TH such as subcutaneous fat necrosis, a benign condition characterized by inflammation and necrosis of subcutaneous fat, and cold panniculitis which is an acute nodular, erythematous eruption. In the TOBY RCT 1% of infants had subcutaneous fat necrosis (204) which can be complicated by hypercalcaemia and require hyperhydration and diuretic treatment (205). Sclerema neonatorum is a diffuse hardening of the subcutaneous tissue during TH that usually self resolves. The use of a gradient variable mode of temperature control has less adverse skin events than automatic servo-controlled mode (206).

In summary, daily skin examination for complications and calcium monitoring during TH is recommended.

## Inborn Errors of Metabolism (IEM)

Many IEM present with NE due to early accumulation of toxic metabolites in the CNS including urea cycle defects, amino acid, and organic acid disorders. IEM of energy deficiency most commonly present in the first few hours and days of postnatal life, with NE, cardiorespiratory compromise and organomegaly, including galactosemia, some organic acidemias, urea cycle defects and fatty acid oxidation defects, whereas inborn errors of intermediate metabolism and substrate usually present later.

Persistent acidosis, hyperlactaemia, and refractory hypoglycaemia may indicate an IEM causing the encephalopathy. Congenital malformations, dysplasias, and dysmorphic features raise suspicion of inherited metabolic disorders in an infant with NE (207). Opisthotonus and myoclonic jerks may distinguish metabolic encephalopathies from other etiologies of NE (208). Full family history of metabolic disorders, sudden infant death, failure to thrive, specialized dietary requirement, developmental delay, and consanguinity may help with the diagnosis.

In the context where an IEM is suspected, Burton et al., recommend serum investigations of blood gas, electrolytes, glucose, ammonia, amino acids, and lactate as well as urinalysis for reducing substances, ketones, amino acids, and organic acids as first line investigations (209). Laboratory findings of lactic acidosis with normoglycaemia may indicate potential medium or long chain fatty acid disorder or glutaric aciduria T2, whereas lactic acidosis with hypoglycaemia may indicate an oxidative phosphorylation disorder. Ketosis with normoglycaemia differential include the organic acidurias, however an acquired metabolic disorder from sepsis and/or dehydration may present similarly (210). Moderately high ammonia levels may be seen in NE without an IEM disorder, with mean levels of 222 μg/dl in one review of infants with NE (211). Higher levels of ammonia is seen in both urea cycle defects are accompanied by respiratory alkalosis and no acidosis and in organic acidemias distinguished by an accompanied metabolic acidosis.

Management of IEM is dependent on the underlying etiology and is done in conjunction with metabolic specialist advice. The overall management includes prevention of accumulation of harmful substances by stopping enteral and parenteral nutrition and correction of metabolic abnormalities by normalizing glucose with IV dextrose, and aiming to normalize blood pH, and eliminate toxic metabolite accumulation (212).

In conclusion; IEM may present as NE, with distinguishing laboratory and or dysmorphic features. Prompt investigation and targeted management of the underlying IEM disorder is necessary. Acquired metabolic disorders secondary to other etiologies of NE may present with laboratory findings of high lactate levels, moderately high ammonia, and hypoglycaemia.

## Multi-Organ Scoring in NE

In view of the multiorgan involvement on NE several groups have developed scoring systems to evaluate organ involvement (Table 1). Shah et al. described multi organ scoring dysfunction in infants with severe NE (*n* = 144) (10). They included renal, pulmonary, cardiovascular, and hepatic parameters. All infants had minimum of one end organ dysfunction. Renal, cardiovascular, pulmonary, and hepatic dysfunction were found to present in 70, 62, 86, and 85% of infants, respectively. They concluded that multi organ dysfunction may be included to support a diagnosis of NE, but that multi-organ dysfunction did not correlate with adverse neurodevelopmental outcomes or death.

**Table 1:** Comparison of definitions of Multi-Organ Dysfunction in Neonatal Encephalopathy.

	**Shah et al. (10)**	**Hankins et al. (177)**	**Martin-Ancel et al. (11)**	**Perlman et al. (213)**	**Alsina et al. (214)**
**Multi-Organ dysfunction in neonatal encephalopathy**					
CVS	Hypotension treated with an inotrope for >24 h to maintain normal BP ECG evidence of transient myocardial ischaemia	Need for inotropic support beyond 2 h post birth Elevated CK-MB isoenzyme	Systolic and or diastolic BP <5th percentile for age and sex ECG abnormalities ECHO abnormalities	ECG or ECHO abnormalities(values > 2 SDs fromthe mean normal values)	Troponin >1 ug/L Need for vasoactive drugs
Hepatic	AST or ALT> 100 IU/l during week one	Elevation of AST, ALT, LDH to 1.5 x upper normal level	Not included	Not included	GOT or GPT >100 Prothrombin activity <60%
Renal	Anuria or oliguria for ≥24 h and a SCr >100 mmol/l Anuria/oliguria for >36 h Any SCr >125 mmol/l Serial SCr increasing postnatally	Elevation of SCr to ≥88.4 mmol/l Oliguria >24 h Persistent haematuria or proteinuria	Oliguria for > 24 h ≥2+ of proteinuria Azotaemia = BUN >7 mmol/l	Urea >7 mmol/l azotaemia SCr >90 mmol/l after post-natal D3 Oliguria (<1 ml/kg/hr) for >24 h	Creatinine >1 mg/dl Diuresis <0.99 ml/kg/h Need for replacement therapy
Neuro	Not included	Clinical evidence of NE EEG abnormalities Neuroimaging abnormalities	Abnormal Neurological exam EEG abnormalities Neuroimaging abnormalities	Abnormal neurological exam Cranial ultrasound abnormalities	Not included
Resp	Need for ventilatory support with 40% oxygen for at least the first 4 h after birth	Not included	Abnormal Silverman Score Need for 0_2_ supplementation Need for Mechanical Ventilation	Requirement for intubation and Mechanical Ventilation > 48 h post birth	Need for resp support due to causes other than central apnoea or pharmacological effects
GI	Not included	Not included	Gastric residuals, vomiting, abdominal distension/tenderness, and GI bleeding	Evidence of NEC	Not included
Haem	Not included	Thrombocytopenia (<100 × 10^9^/L) Increase in nRBCs to ≥26 per 100 WBCs	Not included	Not included	Leucocyte <4.5 or >30 mm^3^ Platelet <149 APTT >45 s Platelet or FFP

*Scoring systems by Shah, Hankins, Martin-Ancel, Perlman, and Alsina assess systems of Cardiovascular, Hepatic, Renal, Neurological, Gastrointestinal, and Hematological Dysfunction*.

*CVS, cardiovascular; Neuro, neurological; resp, respiratory; GI, gastrointestinal; Haem, hematological; BP, Blood Pressure; AST, Aspartate Amino Transferase; ALT, Alanine Amino Transferase; SCr, Serum Creatinine; CK-MB, Creatinine Kinase Muscle-Brain-Type Isoenzyme; ECG, Electrocardiographic; ECHO, Echocardiographic; SDs, Standard Deviations; NE, Neonatal Encephalopathy; EEG, Electroencephalographic; BUN, Blood Urea Nitrogen; 0_2_, Oxygen; GI, Gastrointestinal; MV, Mechanical Ventilation; NEC, Necrotising Enterocolitis; nRBCs, Nucleated Red Blood Cells; WBCs, White Blood Cell; Glutamic oxaloacetic transaminase (GOT) Glutamic pyruvic transaminase (GPT)*.

Martin-Ancel et al. examined multi organ dysfunction in 72 infants with perinatal asphyxia (11) and found the following distribution: pulmonary (26%), cardiac (29%), gastrointestinal (29%), renal (15%), and respiratory (19%). They included infants from mild to severe NE but only 35% of their included infants required NICU admission, suggesting many of the included infants had milder NE. Apgar score was the only perinatal factor that correlated with the degree of multi- organ dysfunction in their review.

Hankins et al. reported liver injury in 80%, cardiac involvement in 78%, and renal injury in 72% in a prospective review (*n* = 46) (126). These scoring systems allow the evaluation of organ dysfunction but have not yet been assessed in conjunction with longer-term neurodevelopmental or multiorgan follow-up in childhood.

## Conclusion

There has been significant progress over the past two decades in neuroprotective strategies and with the establishment of TH as the standard of care in NE. There remains a gap in the full understanding of the optimal management of the infants during TH, and evidenced based multi-organ support. Ongoing RCTs and systematic reviews to gather information to recommend evidence based best practices is essential to establish the most appropriate practices for management of neonatal seizures, fluid status, inotropic support, and respiratory support.

Establishing evidenced based guidelines for managing multi-organ dysfunction in NE during TH can reduce practice variation, optimize management, and contribute to better outcomes. New adjunctive therapies for NE efficacy may be dependent on the adequate functioning of specific end organs. Dysfunction of these end organs may negatively impact on the efficacy of new treatments and conversely new treatments may have possible adverse side effects on already impaired organ functioning. The introduction of specialized neurocritical care units shows promise in non-pharmacological advancements of management of NE.

Further biomarker development and validation is important to aid in diagnosis of organ injury and prediction of long term outcome, with the BEST (Biomarkers, EndpointS, and other Tools) and twenty first Century Cures Act, providing framework and supportive infrastructure for this (215). Development of an overall predictive model which includes multiorgan dysfunction as part of its criteria is vital to furthering our understanding of NE, and will help in the long-term follow up and care of survivors of NE.

## Author Contributions

MO'D: main writer of paper. DS: original idea for paper and table of multi organ dysfunction. EM: supervisor and advisor of paper and also devised and designed the review. SB, TA, and ME-B: experts in area and reviewed and provided advise on subsections.

## Conflict of Interest

The authors declare that the research was conducted in the absence of any commercial or financial relationships that could be construed as a potential conflict of interest.
